# MiR-369-3p participates in endometrioid adenocarcinoma via the regulation of autophagy

**DOI:** 10.1186/s12935-019-0897-8

**Published:** 2019-07-11

**Authors:** Ping Liu, Chengbin Ma, Qiongwei Wu, Wenying Zhang, Cao Wang, Li Yuan, Xiaowei Xi

**Affiliations:** 1 Gynecology Department, Shanghai General Hospital of Nanjing Medical University, Shanghai, 200080 China; 2 Gynecology Department, Changning Maternity and Infant Health Hospital, Shanghai, 200051 China; 3 School of Basic Medical Sciences of Nanjing Medical University, Nanjing, 211166 China

**Keywords:** Endometrioid adenocarcinoma, MicroRNA, Autophagy, ATG10

## Abstract

**Background:**

The aim of this study is to examine miRNA profiling and miR-369-3p participates in endometrioid adenocarcinoma (EEC) via the regulation of autophagy.

**Methods:**

EEC and its adjacent normal tissues were obtained from 20 clinical patients after surgery. MiRNA profiling was performed using next generation sequencing (NGS) and was validated with quantitative real time PCR (qRT-PCR). qRT-PCR was also employed to measure miR-369-3p and autophagy-related protein 10 (ATG10) expression levels. Western blotting assay was performed to measure the expressions of ATG10 and LC3B. Luciferase reporter assay was conducted to confirm the direct targeting of ATG10 by miR-369-3p. Cell proliferation and migration assays were utilized to analyze the role of miR-369-3p in HEC-1-A cells.

**Results:**

We found that miR-369-3p expression levels were down-regulated in EEC compared to the control tissues. The overexpression of miR-369-3p inhibited cell proliferation and migration in EEC; furthermore, ATG10 expression increased in EEC tissues. ATG10 was found to be a potential target of miR-369-3p via a dual-luciferase reporter assay, and ATG10 was shown to be down-regulated by miR-369-3p in protein levels.

**Conclusions:**

This study revealed that miR-369-3p inhibited cell proliferation and migration by targeting ATG10 via autophagy in EEC.

**Electronic supplementary material:**

The online version of this article (10.1186/s12935-019-0897-8) contains supplementary material, which is available to authorized users.

## Background

Endometrial adenocarcinoma (EEC) is a common and highly malignant gynecological tumor [1]. In recent years, its incidence rate in China and abroad has shown an upward trend, with the prognosis of EEC being poor and the improvement of prognosis depending on early diagnosis [2]. However, the early diagnosis of EEC is challenging. Specifically, identifying highly sensitive and specific tumor markers is a challenge that needs to be solved for the early diagnosis of this cancer.

Autophagy is an important catabolic process in which damaged organelles or protein recycling undergoes nutrient starvation or stress [3], and it is required for glucose homeostasis and lung tumor maintenance [4]. Autophagy participates in a critical pathway that maintains intracellular balance in regard to both the initiation and prevention of cancer [5]. It also supports the survival of tumor cells under metabolic and therapeutic stress [6]. Furthermore, the inhibition of autophagy can increase the chemo sensitivity of cancer cells [7, 8]. However, autophagy is a double-edged sword when regarding health and disease [9]. Autophagy begins with autophagosome formation and concludes with a completed autophagolysosome [10]. In addition, the synthesis of the autophagosome complex is regulated by autophagy-related genes (ATG) [11–13]. Several important genes are involved in the autophagy process, including ATG3, ATG5, ATG7, ATG10, ATG12, and LC310. More evidence has also highlighted the importance of these aforementioned autophagy-related genes in cancer maintenance, clinical therapy, and pathogenesis 8. ATG10 is an E2-like enzyme involved in two ubiquitin-like modifications essential to autophagosome formation [14]. Increased expression of ATG10 in colorectal cancer is associated with lymphovascular invasion and lymph node metastasis [15]. Through the genetically engineered mouse models, ATG7 ablation was found to block tumor growth and induce tumor cell death in lung cancer, indicating that autophagy inhibition might serve as a promising strategy for lung cancer therapy [12]. However, the mechanisms and clinical value of these critical regulatory autophagy-related genes have not been explored in regard to EEC.

MicroRNAs (miRNAs or miRs) are classified as small, endogenous, non-coding, single-stranded RNAs that regulate target genes post-transcriptionally by specifically binding to the 3′ untranslated region (3′-UTR) of the target mRNA [16]. It was reported that miRNAs play an important role in various biological processes, including cell growth, cell death, apoptosis, and tumorigenesis [17, 18]. Several studies using miRNA microarray analyses or miRNA-Seq have revealed that the expression of miR-369-3p increases in the skin tissues of psoriasis patients [19] and that the aberrant expression of miR-369-3p correlates with disease severity in hepatocellular carcinoma [20]. Hu et al. [21] reported that miR-369 induced cellular reprogramming and regulated malignant phenotypes of human colorectal cancer. Li et al. found that miR-369-3p was differently expressed in Hirschsprung disease and may play a crucial role in its pathology [22]. Agarwal et al. [23] indicated that the upregulation of miR-369-3p suppresses cell migration and proliferation by targeting SOX4 in Hirschsprung’s disease. However, the detailed mechanism of miR-369-3p and its potential target gene involved in the pathogenesis of endometrial adenocarcinoma is still not widely understood.

Therefore, we sought to determine whether miR-369-3p is involved in the pathology of EEC and whether miR-369-3p contributes to EEC by regulating autophagy.

## Methods

### Experimental subjects

This is a retrospective study of EEC tissues and their adjacent non-cancerous counterparts regarding four patients treated by surgical resection. The EEC patients were admitted to the Shanghai First People’s Hospital from July 2017 to July 2018. None of the patients underwent chemotherapy, radiotherapy, or steroid treatment. This study was approved by the Review Board of the Shanghai First People’s Hospital under the supervision of the Ethics Committee, and all patients signed the informed consent forms. This study’s protocol conformed to the ethical guidelines of the 1975 Declaration of Helsinki. In this study, EEC and their adjacent normal tissues were obtained from 20 clinical patients. Four pairs were used for next generation sequencing (NGS) detection, and the others were used for qRT-PCR or Western blot (WB) detection. Diagnosis of EEC was based upon clinical, imaging, and histopathological findings based on explant evaluation according to the revised WHO classification of tumors. Patients were aged 40–45 years, with a mean age of 43.8 years. The EEC stage, in accordance with the surgery-pathological staging criteria issued in 1988 by the International Federation of Gynecology and Obstetrics (FIGO), was identified as III–IV. Pathological records and tissue samples were collected during surgery and immediately snap-frozen in liquid nitrogen. The clinical characteristics of the patients are presented in Table 1.

**Table 1:** Clinical characteristics of the patients with EEC

Sex (female)	20
Mean age	43.8 ± 1.3
Stage (FIGO)	1a + 1b
TNM Classification	I/II
Nodules metastases	No

### Cell culture

HEC-1-A cells (human EEC cells-1-A) are human EEC cell lines and were obtained from the American Type Culture Collection (Manassas, VA, USA) and cultured in McCoy’s 5A modified medium supplemented with 10% fetal bovine serum and 1% penicillin/streptomycin (Sigma-Aldrich, St Louis, MO, USA). HEC-1-A cells are used as an accepted model for studying endometrial cancer [22]. MiR-369-3p, a mimic, inhibitor, agomir or antagomir, and an agomir negative-treated control (NTC) (final concentration, 2.5 nmol/L) were purchased from RiboBio Inc. (GuangZhou, China). An adenovirus (Ad)-expressing system, used for the overexpression the ATG10 protein (Ad-expression ATG10), was purchased from Hanbio, Shanghai, China, with an Ad-expressing green fluorescent protein (GFP) serving as a control. Cells were treated with miR-369-3p agomir, antagomir, or NTC in six-well plates following manufacture protocol. For the rescue experiments, cells were treated with both miR-369-3p agomir and Ad-expression ATG10.

### MicroRNA sequencing

MiRNAs were isolated using the miRNA kit (Qiagen, Hilden, Germany) according to the manufacturer instructions. The quantity and quality of RNA were assessed using the NanoDrop ND-1000 instrument (Nanodrop Technologies, Wilmington, USA) and the Bioanalyser 2100 system (Agilent Technologies, CA, USA) using the Agilent RNA 6000 Nano according to the manufacturer instructions. MiRNAs were profiled by NGS on the Illumina Hiseq 2000 instrument (Illumina Inc., San Diego, USA) using the Illumina TruSeq Rapid SBS preparation protocol (Arraystar Inc., Rockville, USA). The cDNA library was prepared using the SMART cDNA Library Construction Kit (Clontech Laboratories, Inc. CA, USA) according to the manufacturer instructions. The quantification of the cDNA library was measured using a 2100 Bioanalyser (Agilent Technologies), with the final concentration being 10 pM. Differentially expressed miRNAs between the two groups were analyzed using the two-tailed, homoscedastic *t*-test.

### Validation test of quantitative real time PCR (qRT-PCR)

qRT-PCR was performed to validate the results of the microRNA sequencing. RNA extraction was conducted as previously described. Reverse transcription was conducted using a QuantiTect Reverse Transcription Kit (Qiagen, Germany) according to the manufacturer instructions. The PCR reaction system was created using a total volume of 20 µL containing 10 µL of 2× master mix, 8 µL of diluted cDNA, and 0.5 µL of each of the forward and reverse primers, respectively. The PCR was completed in following steps: step 1, 95 °C for 3 min; step 2, 95 °C for 15 s and 60 °C for 1 min (repeat step 2 for 40 cycles). The GAPDH gene was used as the reference gene, and the 2^−ΔΔCt^ method was used to calculate the relative level of gene expression in EEC and its adjacent non-cancerous counterparts.

### MiR-369-3p target prediction and functional enrichment analysis

mRNA gene targets were identified using TargetScan (v6.2) [24], Miranda, and Microcosm v5 [25]. The Venny tool (Venny v2.0.2) [26] was used to filter miRNA target genes into all three programs. Gene ontology (GO) (TopGO v4.2) grouping categories (Biological Process, Cellular Components, and Molecular Processes) and the Kyoto Encyclopedia of Genes and Genomes (KEGG) pathway analysis (Arraystar Inc., Rockville, USA) were used to perform the functional analysis for predicted miRNA target genes.

### Luciferase assay

The mRNA 3′-UTR, containing the putative target site for miR-369-3p, was amplified from genomic DNA via PCR amplification and inserted into the pMIR-REPORT (RiboBio, Guangzhou, China). A mutant reporter plasmid of the miR-369-3p complementary locus was created with the Quik-Change II Site-Directed Mutagenesis Kit (Stratagene, La Jolla, CA, USA) according to the manufacturer instructions. The HEC-1-A cells were transiently transfected with wild-type or mutant reporter plasmid. Luciferase activity was measured 24 h after transfection using the luciferase activity kit (BioVision, San Francisco, CA, USA) according to the manufacturer instructions. Three independent experiments were performed in triplicate.

### Western blot (WB) assay

The total protein from cells was extracted with RIPA lysate. The homogenates, which contained 20 µg of protein, were run on 12–15% SDS-PAGE gels and then transferred to a PVDF membrane (Millipore, Shanghai, China). The PVDF membrane was blocked with 5% non-fat dry milk for 1 h at room temperature and incubated overnight at 4 °C with primary antibodies, including LC3 (1:300; Abcam), Atg10 (1:1000; GTX), and GAPDH (1:3000; Abcam).

### Cell proliferation

Cell Counting Kit-8 (CCK-8, Dojindo Laboratories, Kumamoto, Japan) was used to detect cell proliferation. HEC-1-A cells treated with miR-369-3p agomir, antagomir, miR-NTC, and Ad-expression ATG10 were briefly seeded into 96-well plates (5 × 10^3^ cell/well) with 100 μl medium. CCK-8 reagents were added into each well at 24 h, 48 h, and 72 h and incubated at 37 °C. The optical density (OD) measured at 450 nm on an enzyme-linked immunosorbent assay reader. Three independent experiments were performed in triplicate.

### Cell migration

24-well culture plates inserted with 8-mm pore size culture (Transwell; Falcon, BD Biosciences) were used to performed the cell migration assays. The lower chamber was filled with 600 μl McCoy’s 5A modified medium with 10% FBS. Cells (1 × 10^5^ cell/well) were seeded to the upper chamber. After 24 h incubation, the number of the bottom well cells were counted by counting chamber.

### Statistical analysis

All statistical analyses were conducted using GraphPad Prism software 5.0 (GraphPad Software, Inc., San Diego, CA). The quantitative data were expressed as the mean ± standard error difference $({\bar{\text{X}}} \pm {\text{SED}}),$ and a paired *t*-test was used for comparisons between the two groups. A value of *p *< 0.05 was considered to be statistically significant. Statistical significance for GO terms was calculated using Fisher’s exact test. The significance of KEGG pathways was determined using the EASE-score, Fisher *p* value, and the Hypergeometric test adjusted for the false discovery rate (FDR).

## Results

### MicroRNA expression profiling involved in EEC samples

To study the full spectrum of miRNAs involved in EEC, small RNA-sequencing was performed, and 296 of the miRNAs were initially detected. Of these, 8 miRNAs were significantly differentially expressed between EEC tissues and their adjacent non-cancerous counterparts. Five miRNAs (hsa-miR-136-3p, hsa-miR-451a, hsa-493-5p, hsa-369-3p, hsa-miR-543) showed down-regulation, and three miRNAs (hsa-miR-10b-5p, hsa-miR-143-3p, and hsa-miR-145-5p) showed up-regulation between EEC tissues and their adjacent non-cancerous counterparts (Table 2).

**Table 2:** Differentially expressed microRNAs identified by next generation sequencing

miRNA information			Statistics and regulation			
Mature-miRNA	Pre-miRNA	Mature-sequence	Fold change	*p*-value	FDR	Regulation
hsa-miR-136-3p	hsa-mir-136	CAUCAUCGUCUCAAAUGAGUCU	63.72	0.005402	0.035995	Down
hsa-miR-369-3p	hsa-mir-369	AAUAAUACAUGGUUGAUCUUU	150.75	0.009838	0.053134	Down
hsa-miR-451a	hsa-mir-451a	AAACCGUUACCAUUACUGAGUU	69.29	0.048753	0.143407	Down
hsa-miR-493-5p	hsa-mir-493	UUGUACAUGGUAGGCUUUCAUU	90.00	0.019385	0.081725	Down
hsa-miR-543	hsa-mir-543	AAACAUUCGCGGUGCACUUCUU	90.40	0.035484	0.117148	Down
hsa-miR-10b-5p	hsa-mir-10b	UACCCUGUAGAACCGAAUUUGUG	3.52	0.012481	0.061076	Up
hsa-miR-143-3p	hsa-mir-143	UGAGAUGAAGCACUGUAGCUC	9.21	4.05E−08	0.000012	Up
hsa-miR-145-5p	hsa-mir-145	GUCCAGUUUUCCCAGGAAUCCCU	2.89	0.012701	0.061076	Up

### Quantitative real time PCR

**Fig. 1:**
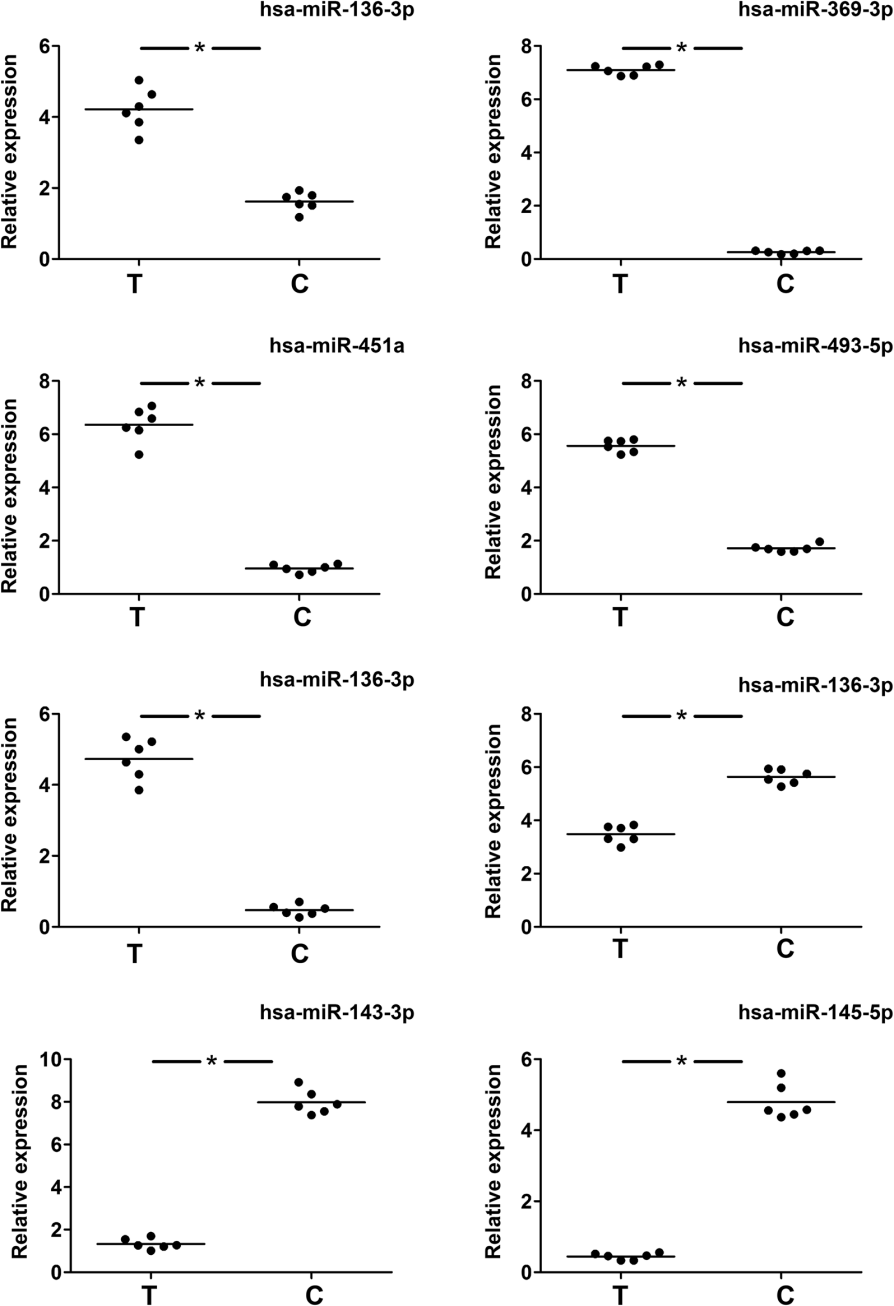
Results of quantitative real time PCR (qRT-PCR). qRT-PCR was used to validate the results of small RNA-Sequencing. The qRT-PCR results demonstrated an increase in the expression of hsa-miR-10b-5p (6.50 ± 0.09-fold), hsa-miR-143-3p (2.70 ± 0.16-fold), and hsa-miR-145-5p (5.60 ± 0.22-fold) in the EEC tissues, whereas the expression of hsa-miR-369-3p (− 7.50 ± 0.24-fold), hsa-miR-136-3p (− 3.72 ± 0.14-fold), hsa-miR-451a (− 2.69 ± 0.13-fold), hsa-miR-493-5p (5.60 ± 0.21-fold), and hsa-miR-543 (− 4.51 ± 0.16-fold) decreased in the EEC tissues, respectively. These data were consistent with the small RNA-sequencing results. T, EEC tissues; C, their adjacent no cancerous counterparts. *p < 0.05

qRT-PCR was then used to validate the results of the small RNA-sequencing. The qRT-PCR results demonstrated an increase in the expression of hsa-miR-10b-5p (6.50 ± 0.09-fold), hsa-miR-143-3p (2.70 ± 0.16-fold), and hsa-miR-145-5p (5.60 ± 0.22-fold) in EEC tissues, whereas the expression of hsa-miR-369-3p (− 7.50 ± 0.24-fold), hsa-miR-136-3p (− 3.72 ± 0.14-fold), hsa-miR-451a (− 2.69 ± 0.13-fold), hsa-miR-493-5p (5.60 ± 0.21-fold), and hsa-miR-543 (− 4.51 ± 0.16-fold) decreased in EEC tissues (Fig. 1). These data were consistent with the small RNA-sequencing results.

### Target prediction and functional enrichment analysis of miR-369-3p

**Fig. 2:**
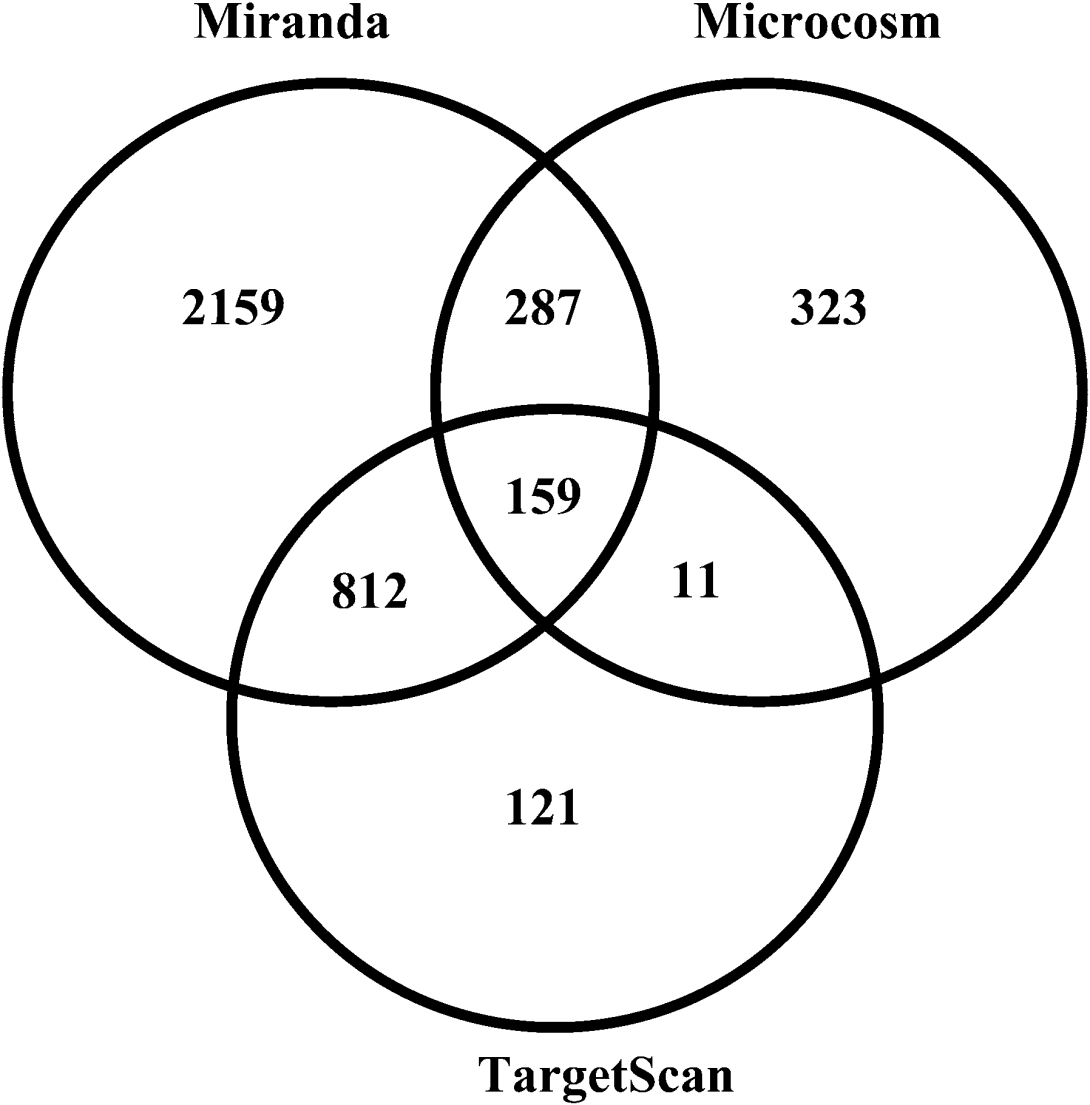
Venn diagram showing mRNA targets for miR-27b predicted using TargetScan, Miranda, and Microcosm. More than 3000 mRNA targets for miR-27b were predicted by each program. Of these, 742 mRNA targets were commonly predicted by TargetScan and Miranda, 261 mRNA targets were commonly predicted by Miranda and Microcosm, 15 mRNA targets were commonly predicted by Microcosm and TargetScan, and 133 mRNA targets were commonly predicted by TargetScan, Miranda, and Microcosm

Over 2500 putative mRNA targets were predicted for miR-369-3p using TargetScan, Miranda, and Microcosm, of which 159 gene targets were commonly predicted by all three programmes (Fig. 2). Moreover, 812 mRNA targets were commonly predicted by TargetScan and Miranda, 287 mRNA targets were commonly predicted by Miranda and Microcosm, and 11 mRNA targets were commonly predicted by Microcosm and TargetScan.

**Fig. 3:**
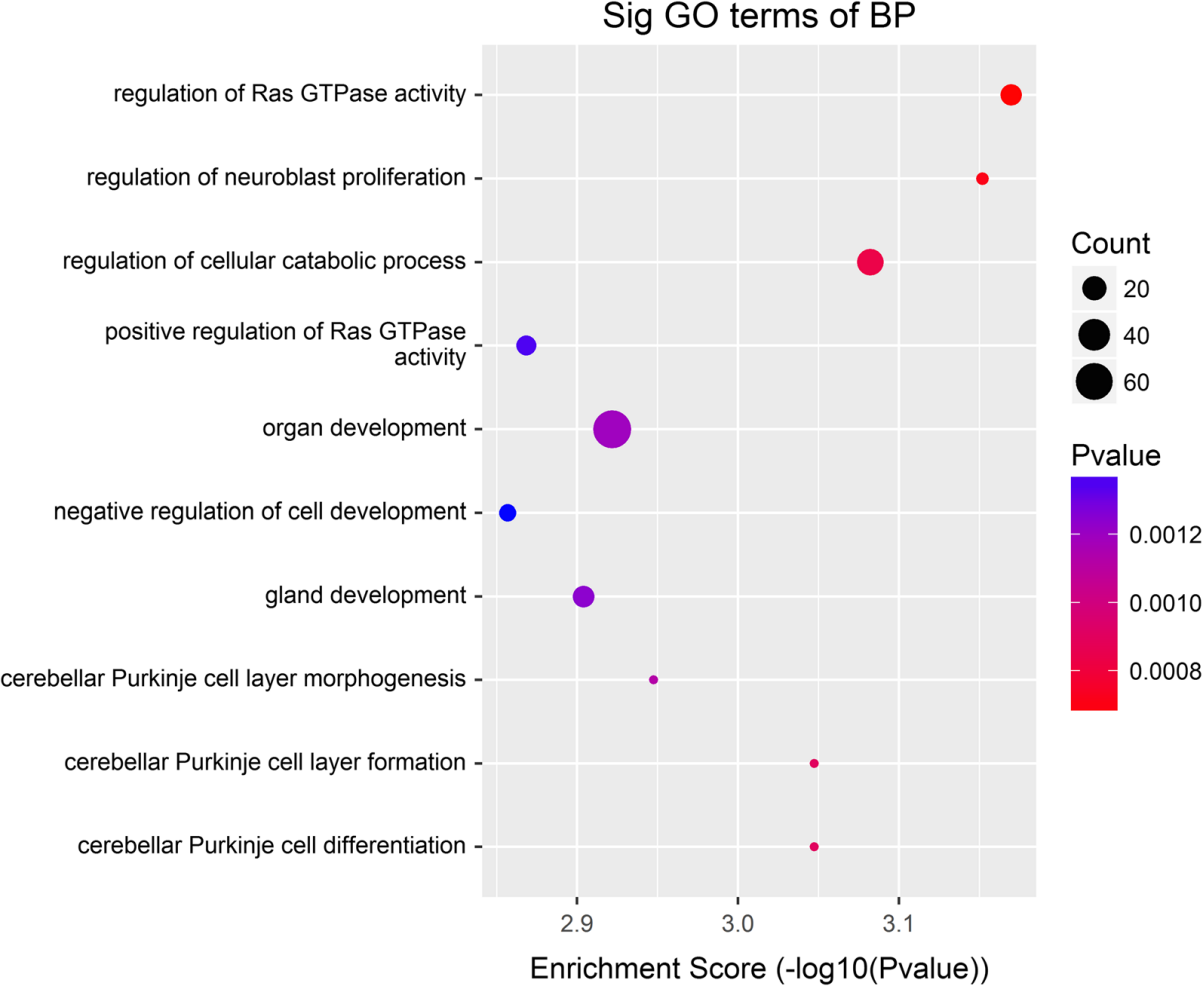
Top ten GO terms enriched by miR-369-3p gene targets. Data are presented as enriched scores expressed as − log10 (p value)

**Fig. 4:**
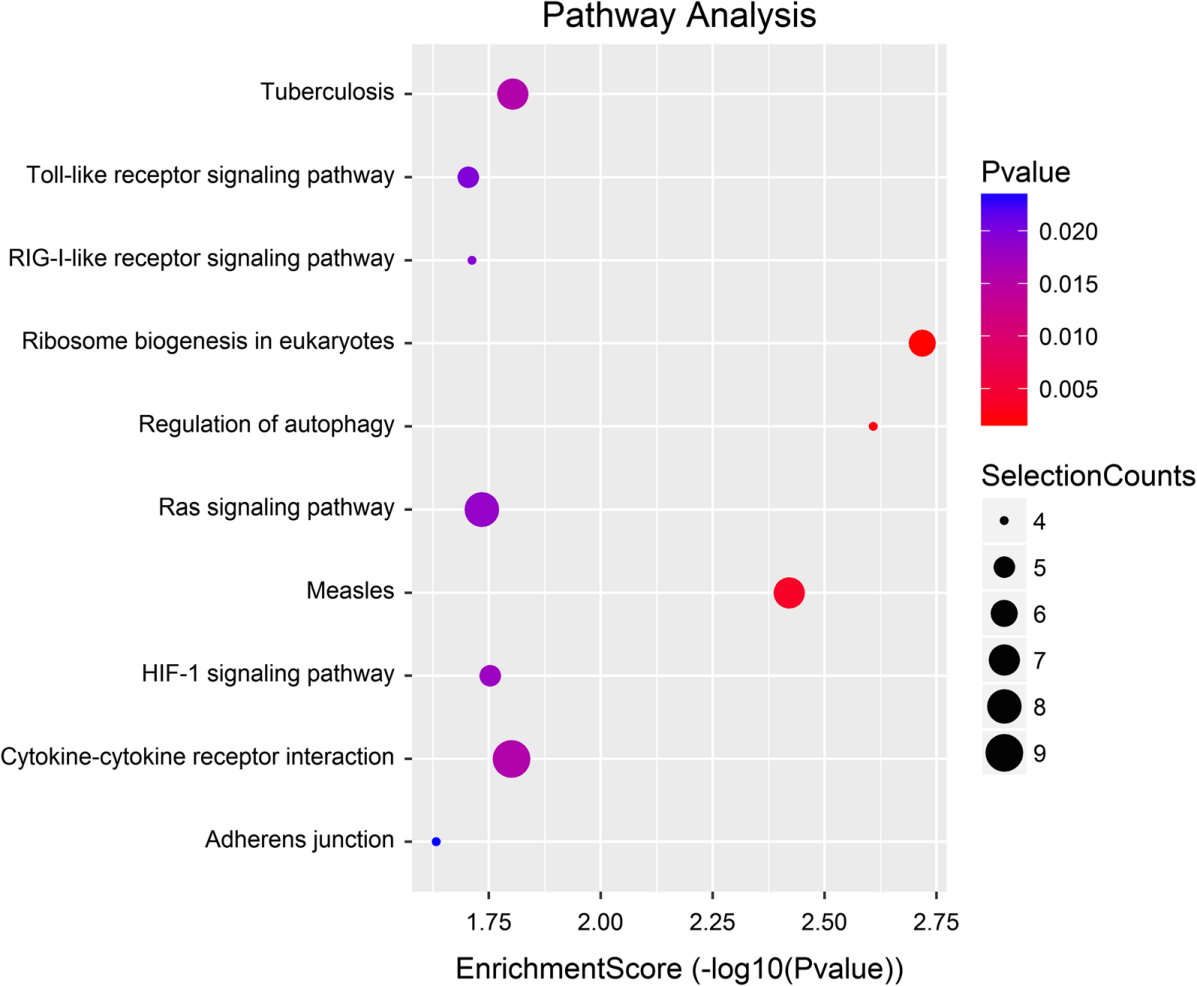
KEGG pathway analysis of miR-369-3p gene targets. MiR-369-3p gene targets were mapped to KEGG pathways. Data are presented as enriched scores expressed as − log10 (p value)

Functional enrichment analysis identified 427 GO terms enriched by miR-369-3p gene targets, categorized into 17 molecular functions (MF), 253 biological processes (BP), and 17 cellular components (CC) (Additional file 1: Table S1), with the top 10 GO terms of BP illustrated in Fig. 3. Three GO terms, (1) regulation of Ras GTPase activity, (2) regulation of neuroblast proliferation, and (3) regulation of cellular catabolic process, were statistically significant with p < 0.001, FDR ≤ 0.51. Five significantly enriched signalling pathways (Fisher p value ≤ 0.05) (Additional file 2: Table S2) were identified. These pathways include the (1) ribosome biogenesis in eukaryotes, (2) regulation of autophagy, (3) measles, (4) tuberculosis, and (5) cytokine–cytokine receptor interaction (Fig. 4).

### Autophagy in EEC tissues

**Fig. 5:**
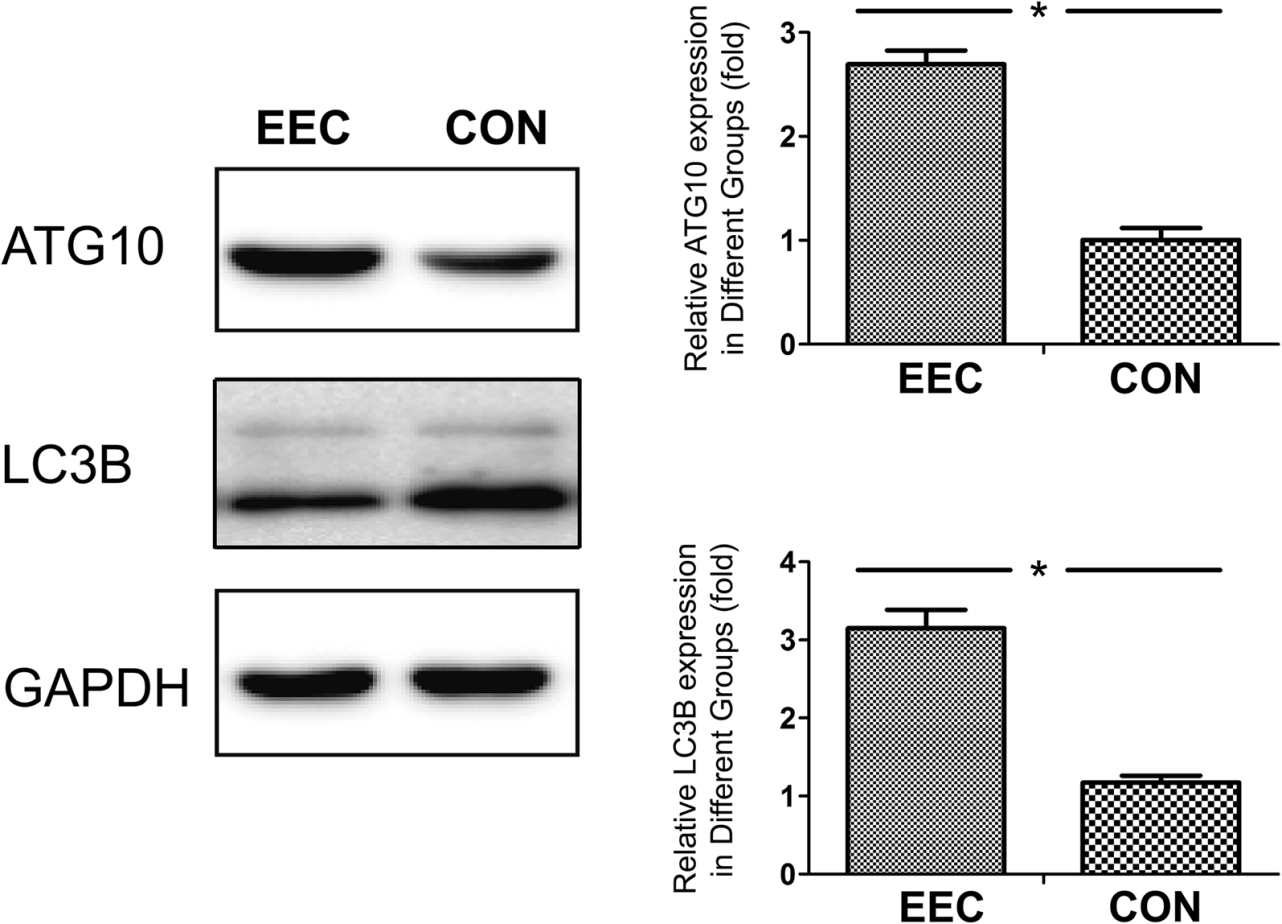
Autophagy in EEC Tissues. Up-regulation of ATG10 and LC3B protein expression levels in the EEC tissues compared with that of their adjacent no cancerous counterparts. Data are expressed as the means ± SED (n = 8 per group). *p < 0.05

In EEC tissues, the expression of ATG10 was significantly upregulated at the protein levels compared to that of its adjacent non-cancerous counterparts. Furthermore, we found that the expression of LC3B was increased more in EEC tissues than in the adjacent non-cancerous counterparts. This increased LC3B indicated that cancer induced the formation of autophagosomes (Fig. 5).

### ATG10 is a direct target of miR-369-3p

**Fig. 6:**
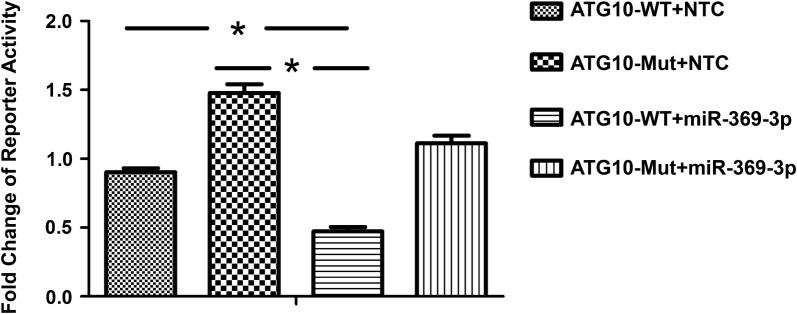
ATG10 Is a Direct Target of miR-369-3p. miR-369-3p targeting to the ATG10 3′UTR was determined by a luciferase reporter assay in HEC-1-A cells. Luciferase activity was measured 24 h after transfection. Data are expressed as the mean ± SED (n = 4 per group). *p < 0.05

To investigate the molecular mechanism of miR-369-3p in endometrioid adenocarcinoma, we employed a bioinformatics approach using TargetScan, Miranda, and Microcosm software to predict the target genes of miR-369-3p. We found that ATG10 may be one of the potential target genes of miR-369-3p. Then, the HEC-1-A cells were treated with miR-369-3p agomir to test the direct link between miR-369-3p and ATG10. We performed a dual-luciferase reporter assay in HEC-1-A cells. After the cells were treated with miR-369-3p, agomir, and the plasmid (pmiR-RBREPORT-ATG10-3′-UTR), a significant decrease in relative luciferase activity was observed (n = 4 per group; Fig. 6). Furthermore, when the complementary sites in the 3′-UTR of ATG10 were mutant, the suppressive effect of miR-369-3p was abolished due to the interaction between miR-369-3p and ATG10 being disrupted. These results suggest the ATG10 is a direct target of miR-369-3p.

### The overexpression of miR-369-3p inhibits EEC cell proliferation and migration via autophagy

**Fig. 7:**
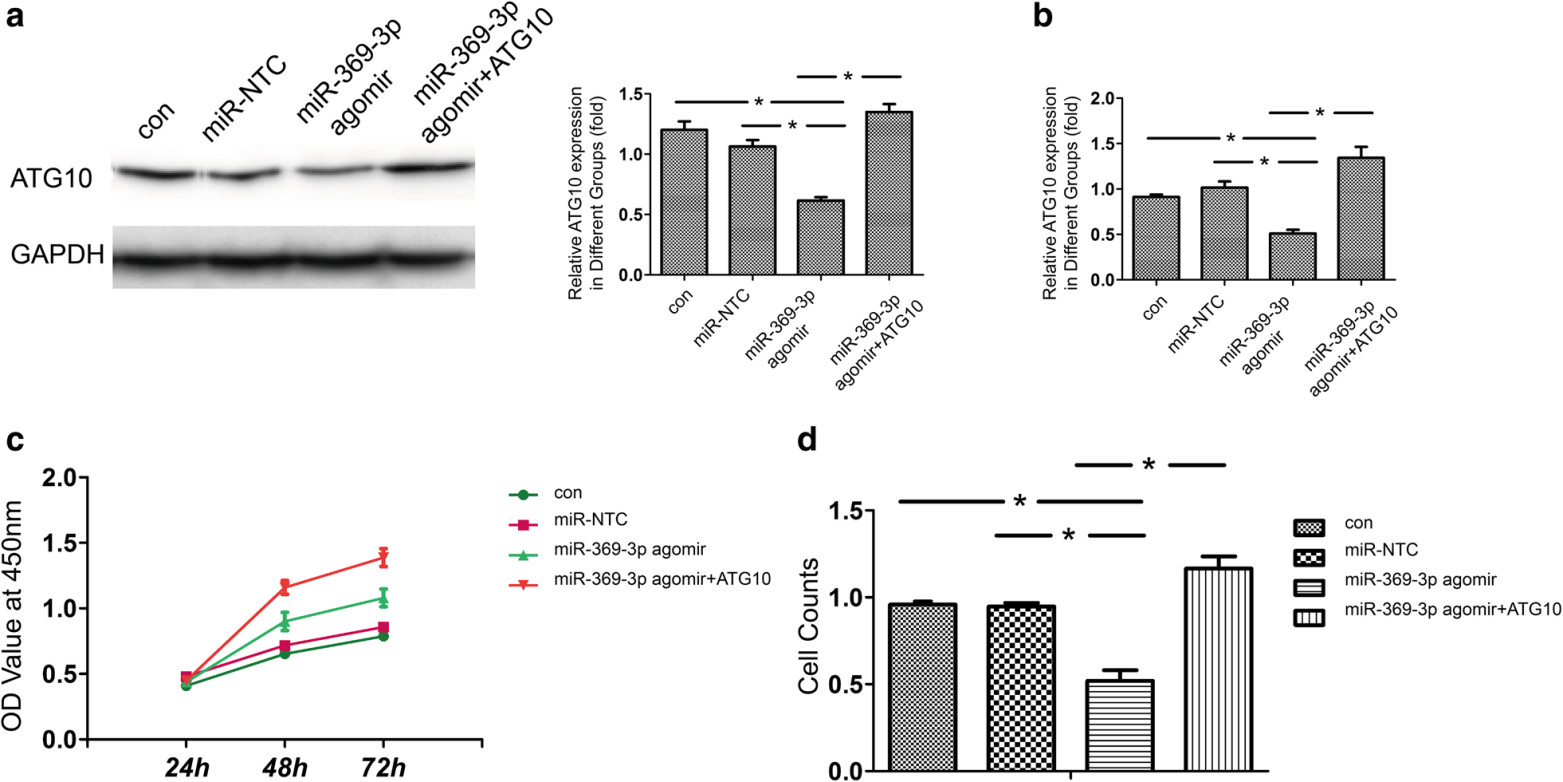
Overexpression of miR-369-3p significantly inhibited HEC-1-A cell proliferation and migration. **a** The treatment with miR-NTC, miR-369-3p agomir and miR-369-3p agomir +ATG10 cells were validated protein expression levels of ATG10 by Western blot. *p < 0.05. **b** The treatment with miR-NTC, miR-369-3p agomir and miR-369-3p agomir +ATG10 cells were validated mRNA expression levels of ATG10 by qRT-PCR. *p < 0.05. **c** CCK-8 assay results showed that overexpression of ATG10 suppressed HEC-1-A cell proliferation. *p < 0.05. **d** Cell migration were detected using transwell assay. Migrated cells were counted by counting chamber in each group. *p < 0.05

Next, we studied whether the overexpression of miR-369-3p can inhibit HEC-1-A cell proliferation and migration by targeting ATG10 in EEC via autophagy. We examined the mRNA and protein expression levels of ATG10 via Western Blot and qRT-PCR after HEC-1-A cell were treated with miR-369-3p agomir or antagomir. Compared with the miR-369-3p agomir group, the mRNA and protein levels of ATG10 were rescued in the miR-369-3p and ATG10 (Fig. 7a, b). Compared to the miR-369-3p agomir group, ATG10 overexpression inhibited cell proliferation (Fig. 7c). Then, we investigated the effects of ATG10 on cell migration in HEC-1-A cells via Transwell assay. The overexpression of ATG10 inhibited cell migration when the cells were treated with miR-369-3p agomir (Fig. 7d). Thus, miR-369-3p promoted cell proliferation and migration by ATG10 through autophagy in HEC-1-A cells.

## Discussion

In this study, we identified miRNA profiling across EEC tissues and their adjacent non-cancerous counterparts. miRNAs from different tissues were identified and compared using NGS and qRT-PCR. Furthermore, we studied the role of miR-369-3p in EEC tissues because (1) miR-369-3p was the most decreased miRNA in the present study and (2) miR-369-3p has been linked to cancerous elements, such as colorectal cancer cells [27] and papillary thyroid cancer [28]. However, there is still little understanding of miR-369-3p in EEC. Using mRNA target prediction analysis, we found and validated that ATG10 was one of the target genes of miR-369-3p. Moreover, we found that ATG10 is a critical gene in autophagy and cancer [29].

New biomarkers for the early diagnosis of the increasing prevalence of EEC requires further development. It was reported that miRNAs play important roles in the occurrence and development of EEC. We found that miR-23a regulates the epithelial-to-mesenchymal transition in EEC by targeting SMAD3 [30]. Lee et al. [31] identified the microRNA expression profile related to lymph node status in women with early-stage grade 1–2 endometrial cancer. Wilczynski et al. [32] reported aberrant microRNA expression in endometrial carcinoma using formalin-fixed paraffin-embedded (FFPE) tissues. In this study, we found that miR-369-3p was significantly downexpressed in EEC tissues compared to control tissues and that the upregulation of miR-369-3p inhibited EEC cell proliferation via ATG10.

Several studies have reported that some miRNAs, such as miR-200c [33] and miR-23a [30], can be used as potential therapeutic approaches to EEC. Furthermore, expression levels of miRNA-200c significantly increased in endometrioid endometrial cancer samples. The expression of miRNA-200c was maintained at significantly higher levels in early-stage endometrioid endometrial cancer compared to more advanced stages. In our previous study, we found that miR-23a expression was down-regulated in human EEC samples, and the overexpression of miR-23a in HEC-1-A cells increased E-cadherin expression while decreasing the expression of vimentin and alpha smooth muscle actin, markers of the mesenchymal cellular phenotype. In this study, miR-369-3p inhibited the proliferation of HEC-1-A cells. Furthermore, miR-369-3p agomir treatment significantly inhibited the growth of HEC-1-A cells. The attenuation of deregulated miR-369-3p expression sensitizes non-small cell lung cancer cells to cisplatin via the modulation of the nucleotide sugar transporter SLC35F5 [33]. It was reported that serum and skin levels of miR-369-3p in psoriasis patients correlated with disease severity [34]. miR-369-3p can inhibit cell proliferation in papillary thyroid cancer by down-regulating the target gene, TSPAN13 [27]. Additionally, LncRNA OIP5-AS1 was reported to regulate radio resistance by targeting DYRK1A via miR-369-3p in colorectal cancer cells [26]. The upregulation of miR-369-3p was also found to suppress cell migration and proliferation by targeting SOX4 in Hirschsprung’s disease [35]. The aforementioned reports suggest that the up-regulation of miR-369-3p can activate multiple signaling pathways required for cancer or other diseases.

## Conclusions

In summary, our data demonstrated that miR-369-3p was downregulated in endometrioid endometrial cancer. Overexpression of miR-369-3p inhibits cancer cell proliferation by targeting ATG10. This mechanism was related to the autophagy signaling pathways.

## Additional files

**Additional file 1: Table S1.** Functional enrichment analysis identified 427 GO terms enriched by miR-369-3p gene targets, categorized into 17 molecular functions (MF), 253 biological processes (BP), and 17 cellular components (CC).

**Additional file 2: Table S2.** Three GO terms, (1) regulation of Ras GTPase activity, (2) regulation of neuroblast proliferation, and (3) regulation of cellular catabolic process, were statistically significant with *p* < 0.001, FDR ≤ 0.51. Five significantly enriched signalling pathways (Fisher *p* value ≤ 0.05) were identified.

## Data Availability

The datasets used and/or analysed during the current study are available from the corresponding author on reasonable request.

## Abbreviations

EEC: endometrioid adenocarcinoma
CCK-8: cell counting kit-8
miR: microRNA
